# Health-related quality of life burden of nonalcoholic steatohepatitis: a robust pragmatic literature review

**DOI:** 10.1186/s41687-018-0052-7

**Published:** 2018-06-19

**Authors:** Tessa Kennedy-Martin, Jay P. Bae, Rosirene Paczkowski, Emily Freeman

**Affiliations:** 1 Kennedy Martin Health Outcomes Limited, 3rd Floor, Queensberry House, 106 Queens Road, Brighton, BN1 3XF UK; 2 Lilly Global Patient Outcomes and Real World Evidence, Indianapolis, IN USA

**Keywords:** Chronic liver disease (CLD), Nonalcoholic steatohepatitis (NASH), Quality of life

## Abstract

**Objective:**

Nonalcoholic steatohepatitis (NASH) is a form of chronic liver disease (CLD): patients have an increased risk of developing cirrhosis, liver failure, and complications (e.g. hepatocellular carcinoma). NASH has a high clinical burden, and likely impairs patients’ health-related quality of life (HRQoL), but there are currently no licensed therapies. The objective of this robust pragmatic literature review was to identify and describe recent studies on the HRQoL burden of NASH from the patient perspective.

**Methods:**

English-language primary research studies were identified that measured HRQoL in adults with NASH (population-based studies or clinical trials of pharmacological therapy). Searches were conducted in the following bibliographical databases: MEDLINE, EMBASE, Cochrane Database of Systematic Reviews (CDSR), Cochrane Central Register of Controlled Trials (CENTRAL), Database of Abstracts of Reviews of Effects (DARE), and Health Technology Assessment Database (HTA). Abstracts from selected congresses (2015/2016) were hand searched. Articles were assessed for relevance by two independent reviewers, and HRQoL data were extracted.

**Results:**

A total of 567 de-duplicated abstracts were identified, and 20 full-text articles were reviewed. Eight studies were included: five quantitative, two interventional, and one qualitative. The quantitative and interventional studies measured HRQoL using the Short-Form 36 (SF-36) and the Chronic Liver Disease Questionnaire (CLDQ), and the qualitative study involved focus groups and individual interviews. Overall, the studies showed that NASH affects HRQoL, especially physical functioning, with many patients reporting being fatigued. In quantitative studies, overall, patients with NASH had a reduced HRQoL versus normative populations and nonalcoholic fatty liver disease (NAFLD) patients, but not versus chronic liver diseases. A longitudinal study showed that when weight loss was achieved, HRQoL improvement over 6 months was greater in patients with NASH versus NAFLD. Qualitative research suggested that, in addition to fatigue, other symptoms are also burdensome, having a broad negative impact on patients’ lives. The impact of pharmacological treatment on HRQoL was explored in only two included studies.

**Conclusions:**

HRQoL is impaired in patients with NASH. Patients experience a range of symptoms, especially fatigue, and the impact on their lives is broad. Further research is needed to understand the HRQoL burden of NASH (e.g. assessing NASH-specific impacts not captured by SF-36 and CLDQ) and the impact of future NASH therapies on HRQoL.

## Background

Nonalcoholic steatohepatitis (NASH) is a form of chronic liver disease (CLD) in adults and children. Histologically, NASH is characterized by hepatic steatosis (fatty infiltration of the liver) and inflammation, with hepatocyte injury (ballooning) with or without fibrosis [1]. Patients have no causes of secondary hepatic fat accumulation, such as excessive alcohol consumption, use of steatogenic medication, or hereditary disorders [1, 2]. In the USA, the estimated prevalence of NASH in the general population ranges from 3% to 5% (reviewed by Vernon et al., 2011 [3]). In obese individuals, the reported prevalence of NASH is up to 56% (reviewed by Lopez-Velazquez et al. 2014 [4] and in Vernon et al., 2011 [3]). Patients with NASH have an increased risk of developing cirrhosis (permanent liver damage, scarring, and dysfunction), liver failure, and complications, such as hepatocellular carcinoma (HCC) [5]. Indeed, NASH is the second leading indication of CLD for liver transplant (LT) in the USA [6]. In a meta-analysis, the rates of liver-specific and overall mortality in patients with NASH were 11.8 and 25.6 per 1000 person-years, respectively [7].

Despite its clinical burden, there are currently no evidence-based licensed therapies for NASH. The World Gastroenterology Organisation (WGO) suggests that, in patients who fail to achieve a 5–10% weight reduction over 6 months to 1 year of lifestyle changes, experimental therapies may be added (such as the antioxidant, vitamin E, and the antifibrotic agent, pentoxifylline) [2]. If these approaches fail, the WGO recommends considering bariatric surgery before the development of cirrhosis. In patients who develop liver failure, LT can be performed; however, this may be denied to patients with morbid obesity, and, even following successful LT, NASH may still recur [2]. Clearly, there is an unmet medical need for novel treatments for NASH that decrease disease progression and clinical impact.

In addition to its clinical burden, NASH likely results in a high patient burden and a negative impact on patients’ health-related quality of life (HRQoL) [8]. Understanding the HRQoL impact of NASH, and the effect of experimental treatments on HRQoL, is important to inform future research on the development of patient-centered outcomes and cost-effectiveness research. In this review, the concept of HRQoL is that of a multidimensional construct, incorporating subjective self-assessment of different domains including physical, emotional, mental, and social functioning in the context of a disease and its treatment [9]. HRQoL is measured using validated HRQoL questionnaires (generic or disease-specific) or is captured through qualitative concept elicitation research with patients. A disease-specific HRQoL questionnaire that is commonly used in patients with CLD is the Chronic Liver Disease Questionnaire (CLDQ) [10]. The CLDQ is a validated 29-item questionnaire that measures HRQoL in six domains (Abdominal symptoms, Fatigue, Systemic symptoms, Activity, Emotional functioning, and Worry), with patients rating the frequency of clinical symptoms and emotional problems with CLD.

The objective of this literature review was to identify and describe studies on the HRQoL burden of NASH from the patient perspective. Studies of interest were: (1) population-based studies (quantitative and qualitative) assessing HRQoL in patients with NASH; and (2) clinical trials assessing the HRQoL impact of (unlicensed) pharmacological therapies for NASH.

## Methods

A search protocol was developed to guide the development and completion of the literature review. It outlined information on the proposed approach, the objective, the search strategy, study selection criteria, data extraction method, and data synthesis methods. The development of a search protocol reduces the impact of review authors’ biases, ensures transparency and accountability, and maximizes the chances of correct data extraction.

### Study selection criteria

Included in the review were English-language primary research studies that measured HRQoL in adults with NASH. Studies could be population-based studies (quantitative and qualitative) or clinical trials of pharmacological therapy. The quantitative measurement of HRQoL could include the use of generic and/or disease-specific HRQoL instruments. Journal articles were included if they were published from 2006 to June 2016, and congress abstracts if they were disclosed at the most recent meetings (2015/2016).

Studies were excluded if they were not specifically in patients with NASH; if they reported an outcome such as disability, depression, or physical functioning as opposed to the multi-dimensional construct of HRQoL; or if they were clinical trials on nondrug treatment (e.g. diet, exercise, or bariatric surgery). Reviews, discussion papers, letters, and editorials were also excluded.

### Information sources

Searches were conducted in in the following bibliographic databases for this review: MEDLINE, EMBASE, Cochrane Database of Systematic Reviews (CDSR), Cochrane Central Register of Controlled Trials (CENTRAL), Database of Abstracts of Reviews of Effects (DARE), and Health Technology Assessment Database (HTA). In addition, the reference lists of identified studies were checked.

The abstracts presented at the most recent meetings of various congresses were searched for relevant studies: International Society for Pharmacoeconomics and Outcomes Research (ISPOR) – both European and international meetings (International 2016; Asia 2016; Europe 2015; Latin America 2015); International Society for Quality of Life Research (ISOQOL) (2015); International Liver Congress, organized by the European Association for the Study of the Liver (EASL) (2016); The Liver Meeting, organized by the American Association for the Study of Liver Diseases (AASLD) (2015); and the Asian Pacific Association for the Study of the Liver (APASL) (2016).

### Search strategy

The literature review is considered to be a robust pragmatic review rather than a systematic review. The approach is robust in that a research plan was developed, a range of bibliographic sources were interrogated, and there was a double abstract review and quality control. However, not all elements were defined a priori, and the review allowed the scope to evolve in terms of focus.

The base search strategy was developed for PubMed, and this syntax was then adapted for the other databases. The search strategies are provided in the Supplement. To remove duplicates, the titles and abstracts of bibliographic records were downloaded and imported into EndNote bibliographic software. The titles and abstracts of the search results were assessed for relevance by two independent reviewers. Studies that met (or could meet) the eligibility criteria were selected for more detailed assessment using the full text. Exclusion codes were assigned at the full text review stage only. In cases of disagreement, both reviewers discussed the record to reach consensus.

Data extraction tables were designed and then populated for all included studies by one reviewer with quality checking undertaken for 10% of records. Information (e.g. study objectives, country, study design, treatments [if relevant], patient population and numbers, HRQoL instruments, and study findings) were included in the data extraction tables.

## Results

**Fig. 1:**
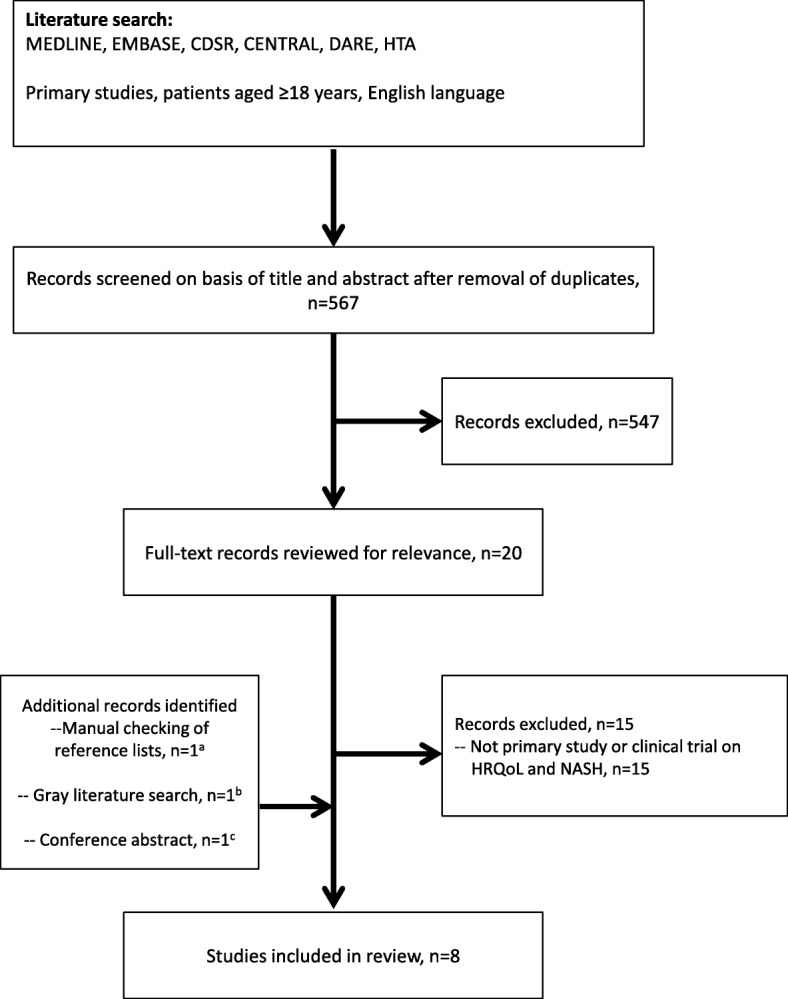
PRISMA flow diagram of search results. ^a^Sanyal et al., 2010 [16] (from an excluded paper). ^b^Sayiner et al. 2016 [13] (from gray searches). ^c^Palsgrove et al. 2016 [17] (conference abstract from ISPOR 2016). HRQoL, health-related quality of life; PRISMA, Preferred Reporting Items for Systematic Reviews and Meta-Analyses

The literature search yielded a total of 567 de-duplicated abstracts, and 20 full-text articles were reviewed for relevance. Three additional records were identified: two from manual checking of reference lists, and one relevant conference abstract. A total of eight studies were suitable for inclusion in the final literature review (Fig. 1). There were five quantitative studies [8, 11–14], two interventional trials [15, 16], and one qualitative study [17]. A summary of the HRQoL -related study objectives and methods is provided below. The HRQoL findings are then described, divided by study type (quantitative, interventional, and qualitative).

### Objectives and methods of the included studies

Table 1 summarizes the study objectives, methods, setting, and HRQoL tools in the eight included studies. All of them were primary research studies that included measurement of HRQoL burden, from the patient perspective, in adults with NASH. The focus of the studies varied: the majority (four of five) of the quantitative studies compared patients with NASH versus other populations (normative, other CLD, nonalcoholic fatty liver disease [NAFLD]), with one study looking at the impact of weight loss on HRQoL. The two interventional studies assessed the impact of treatment on HRQoL and the qualitative study sought to understand NASH-related symptoms and impacts on daily life.

**Table 1:** NASH study objectives, methods, setting, and HRQoL tools/methods in the included studies

Reference	Objective	Study design	Countries	Tools or method of capturing HRQoL	Qualitative analysis software
Quantitative studies reporting HRQoL in patients with NASH					
Alt et al., 2016 [11]	Evaluate HRQoL in patients with CLD and in CLD patients divided by etiology, including NASH	Retrospective, cross-sectional, single-center study	Germany	CLDQ (German version)	IBM SPSS Statistic V.21.0 (IBM Corp., Armonk, NY)
Chawla et al., 2016 [12]	Assess HRQoL in patients with NASH and compare NASH vs normative US data^a^	Prospective, cross-sectional, single clinic study	USA	SF-36, CLDQ	SAS V.8.2 (SAS Institute Inc., Cary, NC, USA)
David et al., 2009 [8]	Compare HRQoL in patients with NAFLD (with and without NASH) vs normative US population	Retrospective, cross-sectional, eight-center study (database and RCT data)	USA	SF-36	NR
Sayiner et al., 2016 [13]	Compare HRQoL in patients with cirrhotic NAFLD vs those with noncirrhotic NAFLD and also vs the general population	Prospective, cross-sectional, single clinic study	USA	SF-36 (and SF-6D health utility scores derived)	SAS (SAS Institute Inc., Cary, NC, USA)
Tapper and Lai, 2016 [14]	Assess HRQoL changes from baseline to 6 months in NAFLD and NASH patients with and without significant weight loss	Prospective, longitudinal (6-month) registry study	USA	CLDQ	JMP Pro statistical discovery (V.11; SAS Institute Inc., Cary, NC, USA)
Interventional trials reporting HRQoL impact of pharmacological therapies for NASH^b^					
Chande et al., 2006 [15]	Determine the effects of an herbal medicine (YHK) versus placebo for 8 weeks on HRQoL (secondary outcome) in patients with NASH and persistently elevated transaminases	Prospective, longitudinal (8-week), randomized, double-blind, placebo-controlled, single-center, pilot study	Japan	SF-36	NR
Sanyal et al., 2010 [16]	Assess the change in HRQoL (secondary outcome) from baseline to the end of 96 weeks’ treatment with pioglitazone, vitamin E, or placebo in patients with NASH (without DM)	Prospective, longitudinal (96-week), randomized, double-blind, placebo-controlled, multicenter, phase 3 clinical trial	USA	SF-36	NR
Qualitative research in patients with NASH					
Palsgrove et al., 2016 [17]	Undertake qualitative research to inform the development of a conceptual model as part of the process of developing a NASH-specific PRO	Qualitative study (focus groups and individual interviews)	North America, Europe, South America, Australasia	Qualitative methods	MAXQDA Plus (VERBI GmbH, Berlin, Germany)

CLD, chronic liver disease; CLDQ, Chronic Liver Disease Questionnaire; HRQoL, health-related quality of life; NAFLD, nonalcoholic fatty liver disease; NASH, nonalcoholic steatohepatitis; NR, not reported; PRO, patient-reported outcome; RCT, randomized controlled trial; SF-36, Short-Form 36; SF-6D, Short-Form Six-Dimension; YHK, Yo Jyo Hen Shi Ko

^a^The primary aim of the study by Chawla et al. (2016) was to assess the reliability and validity of the CLDQ (vs the SF-36) in patients with NASH: CLDQ was found to be a reliable and valid disease-specific HRQoL measure in adults with NASH

^b^There are no licensed therapies for NASH

Two validated HRQoL questionnaires—the generic Short-Form 36 (SF-36) instrument [18, 19] and the disease-specific CLDQ instrument [10]—emerged as frequent choices in the five quantitative studies. The study by Sayiner et al. (2016) [13] also used SF-36 results to derive Short-Form Six-Dimension (SF-6D) health utility scores. The design was cross-sectional in four studies [8, 11–13] and longitudinal in one study [14]. In general, the cross-sectional quantitative studies compared HRQoL in patients with NASH versus normative populations [12, 13], patients with NAFLD [8, 13], and patients with CLDs [11]. The longitudinal study focused on the impact of weight loss (defined as ≥ 5% weight loss) on HRQoL over 6 months in NAFLD patients with and without NASH [14]. The five quantitative studies were conducted in the USA, except for Alt et al. (2016) [11], which was conducted in Germany.

The two interventional trials assessed the HRQoL impact (using SF-36 as a secondary outcome) of unlicensed NASH treatments versus placebo [15, 16]. The trial by Sanyal et al. (2010) [16] was a phase 3 trial called Pioglitazone versus Vitamin E versus Placebo for the Treatment of Nondiabetic Patients with Nonalcoholic Steatohepatitis (PIVENS), conducted in the USA. It compared the change in HRQoL in NASH patients randomized to daily pioglitazone 30 mg (*n* = 80), vitamin E 800 IU (*n* = 84), or placebo (*n* = 83) for 96 weeks. The trial by Chande et al. (2006) [15] was a very small pilot study conducted in Japan that compared the change in HRQoL in NASH patients randomized to an herbal medicine (Yo Jyo Hen Shi Ko [YHK], two 250 mg tablets three times daily) or placebo for 8 weeks.

In the qualitative research study, the objective was to develop a conceptual framework to measure NASH-specific symptoms and impacts [17]. The study was conducted in North America, Europe, South America, and Australasia according to the US Food and Drug Administration (FDA) Patient Reported Outcomes Guidance [20]. Patients with NASH took part in focus groups and individual interviews. From semi-structured discussions (in patients’ native languages), the investigators obtained concepts of NASH-related symptoms and impacts on functioning in daily life. The findings were collated, and the achieved concept saturation was analyzed with qualitative analysis software. Saturation was defined as the point at which no new concepts were noted by subsequent sessions. Finally, concepts were grouped into hypothesized domains, which the study authors plan to use to guide the development of a NASH-specific PRO instrument [17].

### Patient populations in the included studies

Table 2 summarizes the patient populations in the included studies. The patient populations varied among the studies. The number of patients with NASH ranged from 29 [11] to 436 [8] in the five quantitative studies, eight [15] to 247 [16] in the two interventional trials, and was 132 in the qualitative research study [17]. NASH diagnosis included biopsy, with two exceptions. In the study comparing HRQoL in patients with NASH versus patients with other nonviral CLDs by Alt et al. (2016) [11], 81/150 (54%) of the nonviral CLD patients (described below) had biopsy results, but the authors did not state whether this included all 29 NASH patients. The qualitative study enrolled patients with a self-reported NASH diagnosis from a healthcare provider [17].

**Table 2:** Patient populations in the included studies

Reference	Patient population	Patient numbers	How NASH was diagnosed/confirmed
Quantitative studies reporting HRQoL in patients with NASH			
Alt et al., 2016 [11]	Outpatients with noninfectious CLD of different etiologies (including NASH) who were referred to the outpatient clinic of the University Medical Centre of the Johannes Gutenberg University Mainz (excluded malignant disease, previous LT)	CLD *n* = 150 NASH *n* = 29 (19.3%) NAFLD *n* = 25 (16.7%) NASH *n* = 29 Median age 52 y (range 24–76) Males 48.3% Cirrhosis, *n* = 10 (34.5%)	CLD etiology (including NASH) determined clinically and by biopsy Biopsy *n* = 81 (54%)
Chawla et al., 2016 [12]	Adults referred for the evaluation of histology-proven NASH at Mayo Clinic, Rochester (excluded cirrhosis, biliary obstruction)	NASH *n* = 79 Mean age 46 y (SE 11) Males 35% Age-and gender-matched US population sample, *n* = 2474^a^ Mean age 46 y Males 39% Normative data for healthy controls^b^	Biopsy (1) Abnormal serum liver tests for > 3 months; (2) liver histology revealing > 10% steatosis and lobular inflammation with or without fibrosis; (3) exclusion of alternate CLD etiologies; (4) history of alcohol consumption < 40 g/day (men) or < 30 g/day (women); (5) no clinical or biochemical evidence for cirrhosis
David et al., 2009 [8]	Adults with NAFLD (≥ 5% steatosis) and complete biopsy results, from two NASH CRN^c^ studies: (1) the NAFLD Database (observational cohort study); and (2) the PIVENS trial	NAFLD *n* = 713 Definite NASH *n* = 436 (61%) Borderline NASH *n* = 141 (20%) Definitely not NASH *n* = 136 (19%) Definite NASH *n* = 436 Mean age 49.3 y (SD 11.9) Males 33.0% Cirrhosis, *n* = 49 (11.2%)	Biopsy
Sayiner et al., 2016 [13]	Patients with an established histological diagnosis of NAFLD with or without cirrhosis. Patients with viral hepatitis, with significant alcohol intake (> 20 g/day for men, > 10 g/day for women), and with other causes of CLD were excluded	NAFLD with cirrhosis *n* = 30^d^ Mean age 54.1 y (SD 10.8) Males 56.7% NAFLD without cirrhosis *n* = 59 Mean age 49.1 y (SD 10.4) Males 37.3%	Biopsy
Tapper and Lai, 2016 [14]	Patients with NAFLD enrolled in a NAFLD registry (Beth Israel Deaconess Medical Center, Boston, MA) (Excluded other CLDs and daily consumption of > 20 g alcohol)	NAFLD *n* = 151 NASH *n* = 67 (44%) Advanced fibrosis n = 30 (20%) [Age not reported for NASH patients] No significant weight loss achieved *n* = 104 NASH *n* = 42 (44%) Mean NFS –1.41 (SD 1.80) Mean FIB-4 index 1.47 (SD 0.99) Advanced fibrosis *n* = 20 (20%) Significant weight loss achieved *n* = 47 NASH *n* = 25 (54%) Mean NFS –1.95 (SD 1.62) Mean FIB-4 index 1.29 (SD 0.80) Advanced fibrosis *n* = 10 (21.7%)	Biopsy (NAS 5–8)
Interventional trials reporting HRQoL impact of pharmacological therapies for NASH			
Chande et al. 2006 [15]	Adults (18–75 y) with NASH and persistently (≥ 3 months) abnormal ALT and/or AST. (Excluded overuse of alcohol [20 g/week]; other CLDs or other hepatic, GI, renal, cardiovascular, neurological, or hematological disorder; receipt of herbal treatments or dietary supplements except multivitamins/minerals)	NASH *n* = 8 YHK group *n* = 5 Mean age 56 y (SD 7) Males 20% Placebo group n = 3 Mean age 47 y (SD 12) Males 67% Cirrhosis status not stated	Biopsy
Sanyal et al. 2010 [16]	Adults without DM with NASH. Definite or possible steatohepatitis with an activity score of ≥ 5, or definite steatohepatitis with an activity score of 4. A score of ≥ 1 for hepatocellular ballooning was required. (Excluded cirrhosis, other CLDs, alcohol consumption [> 20 g/day women; > 30 g/day men] for ≥ 3 consecutive months during the previous 5 y; receipt of drugs known to cause steatohepatitis)	NASH n = 247 Mean age 46.3 y (SD 11.9) Males 40% Pioglitazone *n* = 80 Vitamin E *n* = 84 Placebo *n* = 83 (Cirrhosis excluded)	Biopsy
Qualitative research in patients with NASH			
Palsgrove et al. 2016 [17]	Patients (≥ 18 y) with NASH, recruited through local clinicians and disease support groups	NASH *n* = 132^e^ Mean age 50.2 y Males 47% Self-reported cirrhosis *n* = 17 (13%)	Self-reported diagnosis of NASH from a healthcare provider

ALT, alanine aminotransferase; AST, aspartate aminotransferase; CLD, chronic liver disease; DM, diabetes mellitus; FIB-4, Fibrosis-4 [index]; GI, gastrointestinal; LT, liver transplant; NAFLD, nonalcoholic fatty liver disease; NAS, NAFLD Activity Score; NASH, nonalcoholic steatohepatitis; NFS, NAFLD Fibrosis Score; PIVENS, Pioglitazone versus Vitamin E versus Placebo for the Treatment of Nondiabetic Patients with Nonalcoholic Steatohepatitis [trial]; YHK, Yo Jyo Hen Shi Ko

^a^Ware JE Jr. SF-36 Health Survey manual and interpretation guide. Boston: The Health Institute, New England Medical Centre, 1993

^b^Bondini S, Kallman J, Dan A, Younoszai Z, Ramsey L, Nader F, Younossi ZM (2007) Health-related quality of life in patients with chronic hepatitis B. Liver Int 27:1119–1125

^c^The multicenter study by David et al. (2009) derived baseline HRQoL data (SF-36) from two studies conducted by the Nonalcoholic Steatohepatitis Clinical Research Network (NASH CRN), comprising a total of eight clinical centers and a central data coordinating center. It was established by the National Institute of Diabetes and Digestive and Kidney Diseases (NIDDK) to assess the natural history, pathogenesis, and therapy of NAFLD in the USA. The first study in David et al. (2009) was a NAFLD Database (observational cohort study), and the second was a randomized controlled trial (Pioglitazone versus Vitamin E versus Placebo for the Treatment of Nondiabetic Patients with Nonalcoholic Steatohepatitis [PIVENS])

^d^Patients with cirrhotic NAFLD in Sayiner et al. (2016) were considered in this literature review to represent NASH

^e^In the qualitative study (Palsgrove et al. 2016), the data in this review have been taken from the conference poster in which the patient number is 132. In the conference abstract, the patient number is 135

There were five single-center studies [11–13, 15, 17], two multicenter studies [8, 16], and one NAFLD registry study [14]. Four of the studies only enrolled patients with NASH [12, 15–17]. Three studies [8, 13, 14] enrolled patients with both NAFLD and NASH. In the NASH CRN study by David et al. (2009) [8], a ‘NAFLD cohort’ included patients from simple steatosis to cirrhosis, and was divided into definite NASH (61% of patients), borderline NASH (20%), or definitely not NASH (19%). HRQoL was compared in ‘NAFLD’ patients with and without definite NASH. In the study by Sayiner et al. (2016) [13], patients with NAFLD were divided into two groups, cirrhotic NAFLD and noncirrhotic NAFLD; those patients with cirrhotic NAFLD were considered in this literature review to represent NASH. In the longitudinal study by Tapper and Lai (2016) [14], 151 patients with NAFLD (*n* = 67 with NASH) were divided at 6 months according to those who achieved significant weight loss (defined as ≥ 5% reduction: *n* = 47; *n* = 25 with NASH) and those without significant weight loss (*n* = 104; *n* = 42 with NASH). As mentioned above, the study by Alt et al. (2016) [11] enrolled patients with nonviral CLDs of different etiologies: cholestatic liver disease (23% of patients), autoimmune hepatitis (21%), NASH (19%), NAFLD (17%), alcoholic liver disease (ALD; 10%), cryptogenic (5%), and ‘other’ (5%).

The rate of cirrhosis in the patients with NASH was generally low. In the quantitative studies, the rate of cirrhosis in NASH patients was zero in Chawla et al. (2016) [12], 11% in David et al. (2009) [8], and 35% in Alt et al. (2016) [11]. Chawla et al. (2016) [12] stated that most of the NASH patients had mild-to-moderate histological involvement with NASH. In Sayiner et al. (2016) [13], as mentioned above, those patients with cirrhotic NAFLD were considered to represent NASH; thus, all the NASH patients had cirrhosis. However, of the cirrhotic NAFLD patients, 73% had mild disease (Child–Turcotte–Pugh class A). The phase 3 interventional trial [16] excluded patients with cirrhosis, and the authors of the small pilot, interventional trial [15] did not report cirrhosis status. In the qualitative research study, 13% of the NASH patients reported that they had cirrhosis [17].

### Quantitative study findings

Overall, the cross-sectional quantitative studies showed that patients with NASH had a reduced HRQoL versus normative populations [12, 13] and versus patients with NAFLD [8, 13]. However, in one study [11], patients with NASH did not have a reduced HRQoL versus patients with CLDs (cholestatic liver disease, autoimmune hepatitis, NAFLD, ALD, cryptogenic, and ‘other’). The findings of these five studies are described in more detail below.

**Fig. 2:**
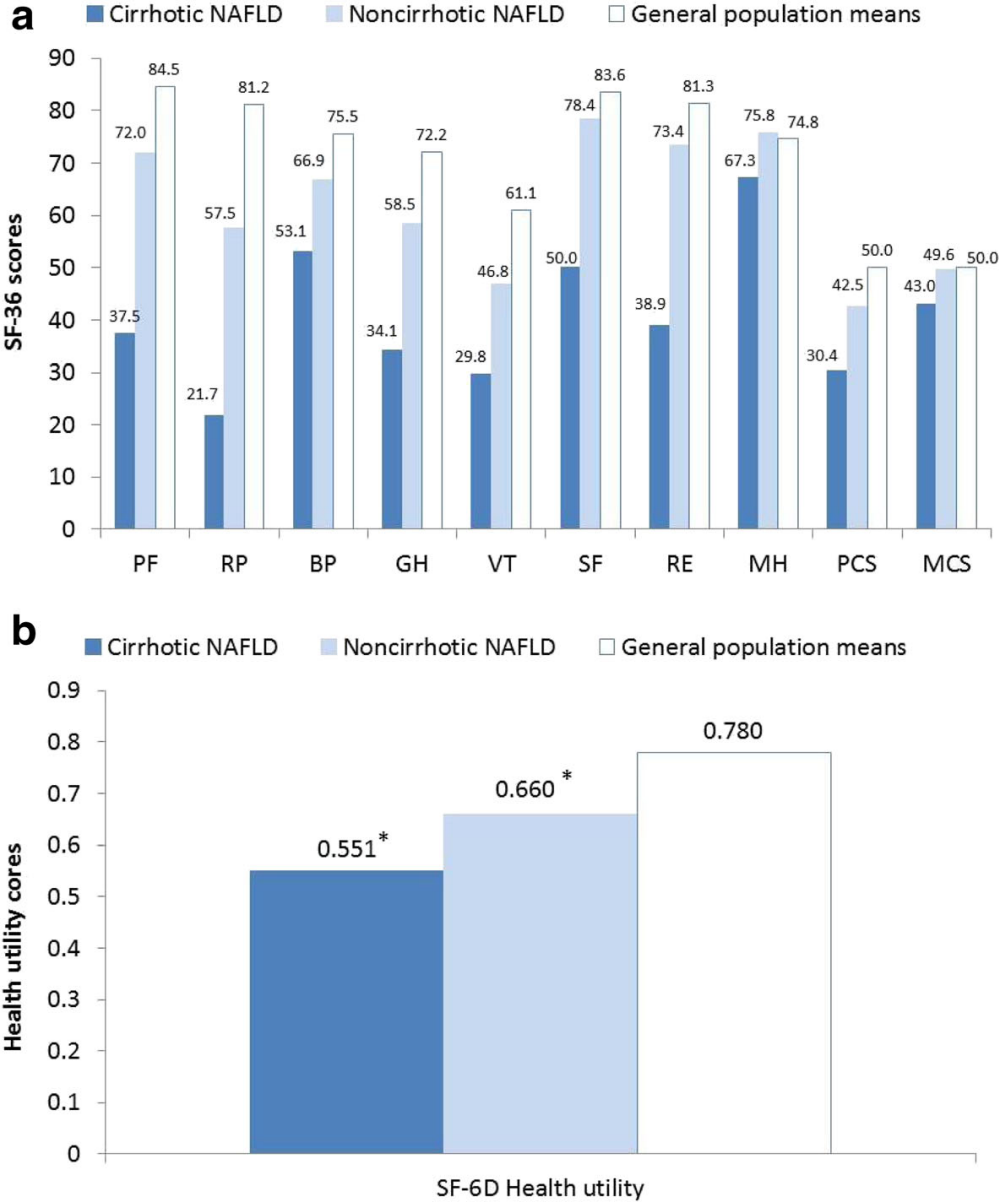
Mean scores for **a** SF-36 domains and **b** SF-6D health utility in cirrhotic NAFLD patients, noncirrhotic NAFLD patients, and general population means (Sayiner et al. 2016) [13]. All domains statistically significantly lower for cirrhotic NAFLD vs noncirrhotic NAFLD (*p* < 0.05), except MH (*p* = 0.0512). Cirrhotic NAFLD: all domains statistically significantly lower versus general population means (*p* < 0.05). Noncirrhotic NAFLD: all domains with the exception of SF, RE, MH, and MCS statistically significantly lower versus general population means (*p* < 0.05). BP, bodily pain; GH, general health; MCS, mental component summary; MH, mental health; PCS, physical component summary; PF, physical functioning; RE, role emotional; RP, role physical; SF, social functioning; VT, vitality. *p < 0.05 versus general population means

In the two studies comparing NASH patients versus normative data, patients with NASH had significantly reduced HRQoL, including generic SF-36 [12, 13] and CLD-specific CLDQ [12] as well as health utilities (SF-6D) [13]. In the study by Sayiner et al. (2016) [13], HRQoL scores were significantly lower in cirrhotic NAFLD patients (considered in this literature review to represent NASH) versus the general population, for SF-36 (all eight domains as well as mental component summary [MCS] and physical component summary [PCS]) and SF-6D health utilities (Fig. 2). In the study by Chawla et al. (2016) [12], patients with NASH had significant impairments in all SF-36 domains versus an age- and gender-matched US population. This was seen in all eight domains (all *p* < 0.05) as well as the PCS and MCS scores (both *p* < 0.02), and in all six CLDQ domains (*p* < 0.0001). A multivariate analysis showed that SF-36 MCS and PCS scores were independent of age, sex, body mass index (BMI; ≥ 30 kg/m^2^, i.e. obesity), and fibrosis stage, but that in patients with type 2 diabetes mellitus (T2DM) there was a significant reduction in the SF-36 PCS score (37 vs 45; *p* = 0.04). Analysis of selected variables showed that CLDQ total scores were independent of age, sex, BMI, and fibrosis stage, but that in patients with T2DM there was a significant reduction in CLDQ total score (4.1 vs 5.1; *p* = 0.01) [12].

In the two studies comparing patients with NASH versus NAFLD [8, 13], SF-36 scores were significantly reduced for all domains, except for the MCS score in the large NASH CRN study by David et al. (2009) [8]. In the NASH CRN study, patients with NASH had significantly lower HRQoL than NAFLD patients without NASH in the PCS score (*p* = 0.018), but not the MCS score (*p* = 0.342) (Table 3) [8]. Scores were also significantly lower for NASH patients in four of the eight domains: Role limitations caused by physical health; Vitality; General health; and Bodily pain (all *p* < 0.05). For the total NAFLD cohort (*n* = 713 including 436 with definite NASH), SF-36 scores were significantly lower than the general healthy US population, for PCS (45.2 vs 55.8) and MCS (47.6 vs 52.5) as well as all domains (all *p* ≤ 0.001). In the NAFLD cohort, the domains with the lowest scores were General health (42.4), Vitality (44.8), and Physical functioning (45.6). In multivariate analysis of the NAFLD cohort, the presence of NASH was not associated with a worse PCS or MCS score [8].

**Table 3:** Standardized mean (SD) SF-36 scores of patients in the NASH CRN with and without NASH (David et al., 2009) [8]

SF-36 domain	All patients^a^ (*n* = 713)	Definite NASH (*n* = 436)	Definitely not NASH (*n* = 136)	*p*-value^b^
Physical Component Summary	45.2 (10.9)	44.5 (11.0)	47.1 (10.4)	0.018
Mental Component Summary	47.6 (11.0)	47.5 (10.9)	48.6 (11.3)	0.342
Physical functioning	45.6 (11.3)	44.9 (11.7)	47.0 (11.0)	0.066
Role limitations due to physical health	46.5 (11.6)	45.8 (11.6)	48.3 (11.4)	0.036
Role limitations due to emotional problems	47.1 (12.2)	46.9 (12.1)	48.6 (12.0)	0.154
Vitality	44.8 (11.2)	44.4 (11.1)	46.6 (11.5)	0.043
Mental health	48.3 (10.8)	48.0 (10.7)	49.1 (11.7)	0.336
Social functioning	46.9 (11.6)	46.9 (11.3)	48.0 (12.0)	0.328
Bodily pain	48.0 (11.2)	47.7 (11.2)	50.0 (11.4)	0.043
General health	42.4 (10.8)	41.8 (10.9)	44.2 (10.9)	0.023

^a^All patients (*n* = 713) includes those with definite NASH (*n* = 436), borderline NASH (*n* = 141), and definitely not NASH (*n* = 136)

^b^ *p*-value is from comparison (t test) between patients with definite NASH and patients with NAFLD but definitely not NASH

An analysis by degree of fibrosis for the total NAFLD population (including NASH patients) found a significant (*p* < 0.001) overall difference in the SF-36 PCS score between fibrosis groups that tended to worsen as fibrosis increased [8]. Fibrosis groups were staged as follows: 0 (none), 1a (mild zone 3 perisinusoidal fibrosis), 1b (moderate zone 3 perisinusoidal fibrosis), 1c (periportal and portal fibrosis [zone 1] only), 2 (both perisinusoidal and periportal or portal fibrosis), 3 (bridging fibrosis), and 4 (cirrhosis). After adjusting for age, sex, race, marital status, education, annual household income, BMI, and T2DM, it was reported that the worsening of fibrosis was independently associated with a significantly worse PCS score compared with no fibrosis (B = − 5.06; *p* < 0.001). If the participants with cirrhosis were removed from analysis, those participants with NAFLD but not cirrhosis had a PCS score of 45.9, which is significantly (*p* < 0.001) lower than the normative reference population. The difference in MCS scores did not significantly differ between the fibrosis categories [8].

In the study by Sayiner et al. (2016) [13], HRQoL scores were significantly (*p* < 0.05) lower in cirrhotic NAFLD patients versus noncirrhotic NAFLD patients for SF-36 (PCS, MCS, and all domains except mental health) and SF-6D health utilities (Fig. 2). In a multiple regression analysis (controlling for age, gender, and ethnicity), presence of cirrhosis was independently associated with lower HRQoL and utility scores in patients with NAFLD. In contrast, there were no independent associations for BMI, cardiovascular disease, or T2DM with HRQoL scores.

The study by Alt et al. (2016) [11] compared HRQoL (CLDQ total scores) in patients with NASH versus patients with other nonviral CLDs. As shown in Table 4, the mean CLDQ overall score was 5.35 (SD 1.1) across the whole CLD population (*n* = 150), and did not differ by CLD etiology, including the 29 patients with NASH (5.28 [SD 1.1]). Although only reported for the total nonviral CLD population, fatigue was the domain that was most affected (CLDQ overall score, 4.61 [SD 1.5]). The main focus of the study by Alt et al. (2016) [11] was to explore the association between hepatocellular apoptosis (measured by serum cytokeratin 18 [CK18]) and HRQoL in these patients. It was found that there was a small negative association between CK18 and HRQoL for total CLDQ score (correlation coefficient [r] = − 0.16; *p* = 0.048) as well in the CLDQ domains Worry (*r* = − 0.21; *p* = 0.01) and Fatigue (*r* = − 0.17; *p* = 0.04). Levels of CK18 were the highest in patients with NASH versus all other CLD etiologies (*p* < 0.001).

**Table 4:** CLDQ overall score (Alt et al., 2016) [11]

Patient group	*n* (%)	CLDQ overall score, mean (SD)
All chronic liver disease	150 (100)	5.35 (1.1)
Autoimmune hepatitis	31 (20.7)	5.28 (1.2)
Cholestatic liver disease	35 (23.3)	5.21 (1.2)
NAFLD	25 (16.7)	5.57 (0.8)
NASH	29 (19.3)	5.28 (1.1)
ALD	15 (10.0)	5.31 (0.9)
Cryptogenic	7 (4.7)	5.86 (0.4)
Other^a^	8 (5.3)	5.39 (1.1)

^a^Other liver diseases included drug induced hepatopathy, haemochromatosis, alpha-1 antitrypsin deficiency, and Budd–Chiari syndrome

In the longitudinal study of weight reduction [14], total CLDQ scores were similar at baseline in NAFLD patients with and without NASH. However, in patients who achieved significant weight loss at 6 months, the associated improvement in CLDQ score was significantly greater in patients with NASH versus those without NASH (Table 5). In contrast, in patients who did not achieve significant weight loss, the change in total CLDQ score was similar in the two groups.

**Table 5:** Changes in CLDQ score by NASH status in patients with and without significant weight loss (Tapper and Lai, 2016) [14]

	Patients with significant weight loss (*n* = 47)		Patients without significant weight loss (*n* = 104)	
Patient subgroup	Change in CLDQ score	*p*-value	Change in CLDQ score	*p*-value
NASH (NAFLD activity score > 4)	0.61 (0.29 to 0.94)	0.0007	0.01 (−0.14 to 0.33)	0.42
No NASH (NAFLD activity score ≤ 4)	0.13 (0.01 to 0.57)	0.04	0.004 (−0.20 to 0.10)	0.97

All values are reported as median (IQR)

Significant weight loss was defined as ≥ 5% reduction at 6 months

### Interventional trial findings

The two interventional trials of patients with NASH did not detect any changes in HRQoL from baseline [15, 16]. In both studies, the PCS and MCS of the SF-36 were presented, but the individual SF-36 domains were not reported.

In the phase 3, 96-week trial [16], PCS and MCS scores did not differ significantly between placebo versus vitamin E or versus pioglitazone (all *p* > 0.05). For PCS, in the placebo, vitamin E, and pioglitazone groups, respectively, mean (SD) scores at baseline were 47 (11), 49 (10), and 49 (9), and the changes from baseline to 96 weeks were − 0.3, 0.4, and − 0.9. For MCS, corresponding mean (SD) scores at baseline were 47 (12), 49 (10), and 49 (8), and the changes from baseline to 96 weeks were 0.4, − 0.5, and − 1.9.

In the small pilot, 8-week trial [15], the effect of the herbal medicine (YHK) versus placebo on both PCS and MCS was variable between the eight patients at each time point (0, 4, 8 weeks of treatment, and 12-week follow-up).

### Qualitative study findings

In the qualitative research study, the development of the conceptual framework provided useful insight on the symptoms that patients with NASH experience, and how these symptoms impact their lives [17]. In the study, focus group and interview participants reported NASH-related symptoms and impacts consistently across 35 sessions. Many symptoms were reported by patients; the most prominent was fatigue, which was experienced by 67% of patients, and reported in 89% of sessions. Other common symptoms were feeling bloated/swollen (35.6% of patients endorsed this), having discomfort/pain around the liver (32.6%), and feeling nauseous/queasy (30.3%).

NASH was associated with a wide-ranging impact on patients, including effects on diet, work, family life, sleep, and usual activities. The most commonly reported impacts were: limits to and frustration with diet (52.3% of patients), reduced or impacted sleep (37.9%), impacts on social/family activities (31.1%), and medication/healthcare frustration (29.5%). An additional theme was ‘fear for the future’, with 26.5% of patients worrying that their condition would get worse [17].

The concepts discussed by patients as either symptoms or impact on activities of daily living were placed in six hypothesized domains: Fatigue, Pain/discomfort, Abdominal issues, Sleep, Social/Emotional issues, and Unclassified concepts (Table 6). From the results, the authors drafted a bifactor framework to guide the creation of a new patient-reported outcomes (PRO) instrument. This consisted of both specific items (e.g. fatigue) and overall traits (e.g. symptoms, impact) [17].

**Table 6:** Domains of the NASH PRO Conceptual Framework in the qualitative study (Palsgrove et al. 2016) [17]

Hypothesized domain	Concepts
Fatigue	Tiredness Weakness Lack of energy Complete as much as you want Exercise/physical activity Complete work or daily activities
Pain / Discomfort	Discomfort from bloating Painful discomfort Pain in stomach
Sleep	Good night’s sleep Sleep without waking
Abdominal issues	Nausea Pressure/tightness Need to pass gas
Social / Emotional	Worry about future Spend time with friends/family Feel frustrated Frustrated by diet
Unclassified	Difficulty concentrating Sweatiness/clammy

## Discussion

This literature review identified a limited evidence base documenting HRQoL in patients with NASH. Eight studies were found to assess HRQoL in patients with NASH and overall, they demonstrate that NASH is associated with impaired HRQoL.

Importantly, most of the patients enrolled in the included studies had not progressed to cirrhosis, and none had progressed to liver failure. As discussed by Chawla et al. (2016) [12], this may limit the generalizability of results. Furthermore, the HRQoL burden is likely to be higher in those NASH patients who have progressed to cirrhosis, HCC, or liver failure. This is important because there is a growing prevalence of NASH-related liver failure, as highlighted by US registry data for LT. NASH is the second leading indication of CLD for LT in the USA [6]. NASH is also the second most common etiology of HCC leading to LT in 2012 [21], and the most rapidly rising indication for simultaneous liver kidney transplantation (2002–2011) [22].

In general, the cross-sectional quantitative studies showed that presence of NASH was associated with a reduced HRQoL versus normative populations [12, 13] and versus patients with NAFLD [8, 13], but not versus patients with other nonviral CLDs [11]. Regarding the comparisons with normative populations, significant impairments in HRQoL were seen in NASH patients across all domains of the SF-36 and CLDQ [12, 13], as well as the SF-6D [13]. The study by Chawla et al. (2016) [12] also found that presence of T2DM significantly reduced both CLDQ total score and the SF-36 PCS score.

Regarding the comparisons to patients with NAFLD, significant impairments in HRQoL were seen in patients with definite NASH as compared to NAFLD but definitely not NASH in most or all of the SF-36 domains: this included the PCS but not the MCS score [8, 13]. A multivariate analysis of the NASH CRN study [8] showed that the presence of NASH was not associated with a worse SF-36 PCS or MCS score. The NASH CRN study [8] is important because it provides data from a large sample of patients in the USA with NAFLD (*n* = 713) and within this with biopsy-proven NASH (*n* = 436). The authors stated that the multicenter design of the NASH CRN and the recruitment of patients from a variety of settings mean that data can be generalized to adults with NAFLD in the USA [8]. It is important to note that the ‘NAFLD cohort’ comprised a range of patients, including those with simple steatosis, fibrosis, NASH, or cirrhosis.

Both the SF-36 and CLDQ have been shown to be reliable and validated in populations with NASH [12], and the studies identified in this review have provided useful information. However, there may be NASH-specific impacts that are not captured by these instruments. The present review identified a qualitative research study by Palsgrove et al. (2016) [17] that was used to inform the development of a conceptual framework on the symptoms that patients with NASH experience and how these impact their lives. The authors state that the data will be used to guide the development of a new PRO instrument for NASH for clinical use and to expand the current research on NASH [17].

Although existing NASH data have limitations, the HRQoL burden of severe liver outcomes, such as cirrhosis, HCC, and LT, has been explored in literature reviews. For example, the multifaceted ways in which a person is impacted by cirrhosis including physical, emotional, social, functioning and economic is detailed in a narrative paper by Loria et al. (2013) [23] and comparative HRQoL data between patients with cirrhosis and noncirrhotic patients used to demonstrate the burden. In a systematic review of 36 articles, patients with HCC had low HRQoL versus the general population (especially physical function, emotional status, and functional ability), versus cancer patients (especially emotional, functional, and social/family well-being), and versus patients with CLD (especially physical aspects) [24].It was suggested that the low HRQoL associated with HCC may be due to the severe symptoms or treatment side effects. In a systematic review of 65 studies, LT recipients had improved HRQoL versus patients awaiting LT (in most HRQoL domains), but still had substantial HRQoL deficits versus healthy controls, especially in physical functioning [25].

Despite the clinical and HRQoL burden associated with NASH, there are currently no licensed therapies for NASH [2]. It is important that novel treatments are developed for NASH that decrease disease progression and reduce the HRQoL impact on patients, with a focus on meaningful outcomes relevant to patients. A number of therapies are being investigated for the treatment of NASH, including insulin sensitizers (thiazolidinediones, e.g. pioglitazone), the antioxidant vitamin E, lipid-lowering drugs, antifibrotic agents (e.g. pentoxifylline), and angiotensin receptor blockers [26]. It will be important to investigate the HRQoL change associated with these NASH treatments, in addition to assessing efficacy and safety. The present review identified two prospective, interventional randomized, controlled trials (RCTs) of (unlicensed) treatments for NASH. Neither study detected any change in SF-36 (PCS and MCS) from baseline. Of note, these trials did not use a disease-specific HRQoL measure, which may have been more responsive to any differences. To the authors’ knowledge, there is one ongoing phase 3 clinical trial of patients with NASH that includes HRQoL assessment. The RESOLVE-IT trial (ClinicalTrials.gov identifier, NCT02704403) is comparing the efficacy and safety of elafibranor versus placebo in patients with NASH and fibrosis. The change in HRQoL (SF-36) is a secondary outcome that will be reported at Week 72 and at the end of long-term treatment (estimated to be 4 years). Primary outcomes include histological improvement, all-cause mortality, and liver-related clinical outcomes. The trial is estimated to complete in December 2021.

A number of limitations of the present review should be noted. The review highlighted that there appears to be a limited amount of published literature on the HRQoL burden of NASH, and the HRQoL impact of treatment of NASH. The search strategy excluded clinical trials on nondrug treatment (e.g. diet, exercise, or bariatric surgery) as well as non-English papers, and journal articles published before 2006, and it cannot be ruled out that other relevant studies may have been published. It is also possible that some clinical trials of treatments for NASH that included an assessment of HRQoL did not make mention of this in the abstract (or title) or were not indexed and were therefore not identified. In addition, a more recent search may reveal further relevant studies.

Another limitation of this review is the difficulties defining and diagnosing NAFLD and NASH. For example, in the included study by Sayiner et al. (2016) [13], patients with NAFLD were divided into cirrhotic NAFLD and noncirrhotic NAFLD, with cirrhotic NAFLD considered in this literature review to represent NASH. However, patients with NASH may not necessarily be diagnosed with cirrhosis. According to Machado and Cortez-Pinto (2014) [27], NAFLD represents a spectrum of liver disease, ranging from simple steatosis to NASH, which can have different degrees of fibrosis and progress to cirrhosis, end-stage liver disease and complications (e.g. HCC).

In addition, it should be noted that in the quantitative studies it is possible that the HRQoL impairment associated with NASH could be due to factors other than the condition itself, for example the presence of comorbidities. Although some of the studies did consider sex-matched populations, and multiple regression analysis was undertaken in two of the studies, there is certainly scope for further in-depth analysis. Depending on the study design, there is a possibility for comorbidity to confound the HRQoL results. As such, it is recommended that future studies consider comorbidity as an important factor, and employ designs and methods to better control for potential confounding due to comorbidities.

Further research is needed to understand the HRQoL burden of NASH and the impact of treatments on patients’ HRQoL. Longitudinal studies are needed to better understand HRQoL burden over time (e.g. from diagnosis of NASH to clinical event). Four of the five quantitative studies in the present review had a cross-sectional design. As discussed by David et al. (2009) [8], cause and effect cannot be attributed: for example, it is not possible to ascertain if poor physical functioning contributes to NASH, or if the presence of NASH results in poor physical function. Future studies could also assess HRQoL across different geographies and patient populations. It would be interesting to assess HRQoL in patients with NASH as the disease progresses to cirrhosis, HCC, and liver failure. Importantly, there is a need for the impact on HRQoL to be measured for future therapies, in addition to capturing histologic and clinical parameters. Such studies should use a disease-specific measure and report the change in HRQoL by treatment response, to assess the association between NASH improvement and HRQoL. Finally, it will be important to determine the performance of the new NASH-specific HRQoL instrument that is in development by Palsgrove et al. (2016) [17].

## Conclusion

In summary, this literature review shows that, although there is a limited amount of published literature on the HRQoL burden of NASH, the available studies do offer some very useful insights. The instruments that have been used to measure HRQoL in studies are the SF-36 and the CLDQ. Although few in number, the quantitative studies consistently showed that HRQoL is impaired in patients with NASH, especially in terms of physical functioning, with many patients reporting being fatigued. The qualitative research suggests that a range of other symptoms are also burdensome, having a broad negative impact on different aspects of patients’ lives. In this review, the impact of pharmacological treatment on HRQoL was explored in only two included studies.

## Abbreviations

AASLD: American Association for the Study of Liver Diseases
ALD: alcoholic liver disease
APASL: Asian Pacific Association for the Study of the Liver
BMI: body mass index
BP: bodily pain (SF-36 domain)
CDSR: Cochrane Database of Systematic Reviews
CENTRAL: Cochrane Central Register of Controlled Trials
CK18: cytokeratin 18
CLD: chronic liver disease
CLDQ: Chronic Liver Disease Questionnaire
DARE: Database of Abstracts of Reviews of Effects
DM: diabetes mellitus
EASL: European Association for the Study of the Liver
FDA: Food and Drug Administration
GH: general health (SF-36 domain)
GI: gastrointestinal
HCC: hepatocellular carcinoma
HRQoL: health-related quality of life
HTA: Health Technology Assessment (database)
IQR: interquartile range
ISOQOL: International Society for Quality of Life Research
ISPOR: International Society for Pharmacoeconomics and Outcomes Research
LT: liver transplant
MCS: mental component summary (SF-36 domain)
MH: mental health (SF-36 domain)
NAFLD: nonalcoholic fatty liver disease
NAS: NAFLD Activity Score
NASH CRN: Nonalcoholic Steatohepatitis Clinical Research Network
NASH: nonalcoholic steatohepatitis
NFS: NAFLD Fibrosis Score
PCS: physical component summary (SF-36 domain)
PF: physical functioning (SF-36 domain)
PIVENS: Pioglitazone versus Vitamin E versus Placebo for the Treatment of Nondiabetic Patients with Nonalcoholic Steatohepatitis [trial]
PRISMA: Preferred Reporting Items for Systematic Reviews and Meta-Analyses
PRO: patient-reported outcome
RCT: randomized controlled trial
RE: role emotional (SF-36 domain)
RP: role physical (SF-36 domain)
SD: standard deviation
SF: social functioning (SF-36 domain)
SF-36: Short-Form 36 (questionnaire)
SF-6D: Short-Form Six-Dimension
SS: Systemic symptoms (CLDQ domain)
T2DM: type 2 diabetes mellitus
VT: vitality (SF-36 domain)
WGO: World Gastroenterology Organisation
YHK: Yo Jyo Hen Shi Ko
